# Machine Learning to Improve Orientation Estimation in Sports Situations Challenging for Inertial Sensor Use

**DOI:** 10.3389/fspor.2021.670263

**Published:** 2021-08-03

**Authors:** Marit P. van Dijk, Manon Kok, Monique A. M. Berger, Marco J. M. Hoozemans, DirkJan H. E. J. Veeger

**Affiliations:** ^1^Department of Biomechanical Engineering, Delft University of Technology, Delft, Netherlands; ^2^Department of Artificial Intelligence, Vrije Universiteit Amsterdam, Amsterdam, Netherlands; ^3^Delft Center for Systems and Control, Delft University of Technology, Delft, Netherlands; ^4^Centre of Expertise Health Innovation, The Hague University of Applied Sciences, The Hague, Netherlands; ^5^Department of Human Movement Sciences, Vrije Universiteit Amsterdam, Amsterdam, Netherlands

**Keywords:** madgwick filter, inertial measurement unit, orientation estimation, kinematics, sports, machine learning

## Abstract

In sports, inertial measurement units are often used to measure the orientation of human body segments. A Madgwick (MW) filter can be used to obtain accurate inertial measurement unit (IMU) orientation estimates. This filter combines two different orientation estimates by applying a correction of the (1) gyroscope-based estimate in the direction of the (2) earth frame-based estimate. However, in sports situations that are characterized by relatively large linear accelerations and/or close magnetic sources, such as wheelchair sports, obtaining accurate IMU orientation estimates is challenging. In these situations, applying the MW filter in the regular way, i.e., with the same magnitude of correction at all time frames, may lead to estimation errors. Therefore, in this study, the MW filter was extended with machine learning to distinguish instances in which a *small* correction magnitude is beneficial from instances in which a *large* correction magnitude is beneficial, to eventually arrive at accurate body segment orientations in IMU-challenging sports situations. A machine learning algorithm was trained to make this distinction based on raw IMU data. Experiments on wheelchair sports were performed to assess the validity of the extended MW filter, and to compare the extended MW filter with the original MW filter based on comparisons with a motion capture-based reference system. Results indicate that the extended MW filter performs better than the original MW filter in assessing instantaneous trunk inclination (7.6 vs. 11.7° root-mean-squared error, RMSE), especially during the dynamic, IMU-challenging situations with moving athlete and wheelchair. Improvements of up to 45% RMSE were obtained for the extended MW filter compared with the original MW filter. To conclude, the machine learning-based extended MW filter has an acceptable accuracy and performs better than the original MW filter for the assessment of body segment orientation in IMU-challenging sports situations.

## Introduction

In sports, inertial measurement units are often used to measure the orientation of human body segments (Camomilla et al., 2018). When an inertial measurement unit (IMU) is attached to a body segment, the orientation of the segment relative to the earth field can be estimated using an orientation estimation algorithm. Although optical motion capturing is widely accepted as a reference system for kinematic measurements, IMUs are often preferred over optical motion capture systems, since they are generally small, wearable (wireless), cheap, and easy to use outside the laboratory. IMU accuracy has shown its validity for several applications in sports (Ahmadi et al., 2010; Bergamini et al., 2013; Le Sage et al., 2013; Najafi et al., 2015; Miller and Kaufman, 2019; Brouwer et al., 2020). Some examples of this are upper body orientations during walking and running on a treadmill (Miller and Kaufman, 2019), pelvis orientation during swimming (Le Sage et al., 2013), and trunk orientation during sports motions that last for only short time periods (<30 s) such as sprint starts, tennis serves, and the golf swing (Ahmadi et al., 2010; Bergamini et al., 2013; Najafi et al., 2015; Brouwer et al., 2020).

An IMU generally consists of an accelerometer, a gyroscope, and a magnetometer, which measure the three-dimensional (3D) linear accelerations (including gravity), the angular velocity, and the local magnetic field, respectively. To estimate the orientation from these raw IMU signals, the data can be fused together using an attitude and heading reference system (AHRS). A commonly used AHRS method is to first obtain two different orientation estimates that are subsequently combined. First, the orientation of the IMU is estimated by integrating the angular velocity, based on the gyroscope signals. As this orientation estimate is distorted by integration drift, the gyroscope-based orientation is “corrected” using a second orientation estimate; the IMU orientation estimated relative to the direction of gravity (down; based on the accelerometer) and the direction of the magnetic field of earth (north; based on the magnetometer). The second estimate will be referred to as the “earth frame-based” orientation estimate. To estimate the earth frame-based orientation, it is assumed that the accelerometer only measures gravity and that the magnetometer only measures the magnetic field of the earth, such that the orientation of the sensor relative to the earth frame, i.e., down and north, is obtained.

In many applications, this assumption can be made, since linear accelerations in directions other than gravity are much smaller than gravity, such that they have a negligible effect on the direction of the acceleration vector. However, in sports activities characterized by relatively large and continuously present linear accelerations (e.g., every push in speed skating or in wheelchair propulsion) or by the presence of close magnetic sources (e.g., from a bike or a wheelchair), the accelerations in directions other than gravity affect the direction of the acceleration vector and the close magnetic sources affect the direction of the local magnetic field. Therefore, during these sports activities, the earth frame-based orientation estimate is often incorrect such that the integration drift is corrected in the wrong direction. In this study, such sports activities are referred to as “IMU-challenging sports situations.”

Some studies solve this problem by combining data of the IMU sensors with that of other sensor types such as force sensors or GPS (Brodie et al., 2008; Zhang et al., 2016). These sources provide additional (indirect) information about the sensor orientation. Another (magnetometer-free) solution that is used to detect the direction of gravity without assuming that only gravity is measured is to attach multiple sensors on connected body segments (Lee and Jeon, 2019; Weygers et al., 2020). Although those approaches previously produced accurate orientation estimates (Brodie et al., 2008; Zhang et al., 2016), the benefits of using a single sensor (easy to use and cheap) diminish. Obtaining accurate estimates based on one or two IMUs only is, therefore, preferred.

To implement the algorithms in already existing sports applications (e.g., smartphones or sports watches) and to enable real-time orientation estimations, the computational efficiency of the algorithms is of interest. A computationally efficient filter that previously provided accurate results in sport settings based on IMU signals only is the Madgwick (MW) filter (Madgwick et al., 2011). The MW filter is resilient against short-term disturbances (Kok and Schon, 2019) and is widely used in sports settings. The filter combines the gyroscope-based estimate and earth frame-based estimate by correcting the gyroscope estimate in the direction of the earth frame-based estimate at each time instance. In this way, the filter corrects for integration drift. The magnitude of this correction, or correction size, is the same at each time instance, and its value is, therefore, crucial to performance (Madgwick et al., 2011). Since the optimal correction size depends on the extent to which integration drift is expected (which depends on the sensor used and the nature of the measurements, i.e., static or dynamic Madgwick et al., 2011), the correction size should be determined for each sensor and application. Commonly, the correction size is determined based on the smallest difference with a reference system and is maintained henceforth (Madgwick et al., 2011; Brouwer et al., 2020).

To obtain accurate orientation estimates in IMU-challenging sports situations, applying a MW filter in the regular way, i.e., with the same correction size at all time frames, will lead to estimation errors. During these sports situations, the correction will be too small to correct for drift or too large such that drift is corrected in the wrong direction (because of a wrong earth frame-based orientation estimate). Therefore, during instances in which the earth frame-based orientation estimate is likely to be wrong, it may be beneficial to temporarily decrease the correction size, i.e., limit the impact of the earth frame-based estimate. In line with this, the correction size should be increased again when the earth frame-based orientation estimate is correct, such that the drift can be controlled. Adapting the correction size in this way has already led to improved orientation estimates in (aerial) vehicles (Yoo et al., 2011; Valenti et al., 2015). However, these studies only took the effect of acceleration into account and implemented self-designed filters. To ensure usability in sports settings, we present a proof of concept in which the widely used MW filter is extended with an adaptive correction size to make it applicable in IMU-challenging sports situations. Since the combined effect of linear accelerations and magnetic sources is expected to be indirect and non-linear, machine learning was used to predict the right time instances for each correction size.

In this study, the MW filter is extended with machine learning to distinguish instances in which a *small* correction size (in the direction of the earth frame-based orientation estimate) is advantageous from instances in which a *large* correction size is advantageous, to eventually arrive at accurate body segment orientation in IMU-challenging sports situations. To this end, a machine learning model was trained to classify whether or not the earth frame-based estimate is likely to be correct based on raw IMU data. Experiments were performed to assess the validity of the extended MW filter, and to compare the extended with the original MW filter. The experiments involved indoor wheelchair sport activities. The presence of a wheelchair and the accelerate–decelerate nature of this sport makes it a representative IMU-challenging sport situation. During wheelchair propulsion, trunk motion is used to prevent the chair from tipping over during large accelerations and may be used to increase stroke length. In addition, trunk motion causes continuous displacements of the center of mass such that, e.g., rolling resistance is affected. Therefore, trunk motion is expected to have a significant role in wheelchair propulsion. Since wheelchair kinematics, such as speed and rotational speed, can already be measured accurately using IMUs in wheelchair match settings (van der Slikke et al., 2015), adding instantaneous IMU-based trunk motion would result in more information about the wheelchair-athlete interaction, which is beneficial for training purposes.

The aim of this study was to investigate whether machine learning-based classification could be used to extend the existing MW filter and, in this way, improve the obtained body segment orientation in IMU-challenging sports situations.

## Methods

### Procedure

Eleven differently skilled participants (Table 1) performed a series of wheelchair sport-specific activities with IMUs attached to their wheelchair and trunk while simultaneously being measured with an optical motion capture (MOCAP) analysis system to serve as reference system. Video recordings were made to distinguish between different activities afterward. The experiment was approved by the ethical committee of the Technical University of Delft. Prior to the experiment, the participants were informed about the aim and procedure of the study and provided a written informed consent.

**Table 1:** Subject characteristics (mean ± standard deviation).

**Type**	***N***	**Age (years)**	**Class^a^**
Elite wheelchair athlete^b^	3	25.0 ± 3.0	3.2 ± 1.3
Active wheelchair user	3	46.3 ± 11.0	2.5 ± 0.5
Non-experienced user	5	25.0 ± 1.2	–

a *The classes were indicated by the points as used in (elite) wheelchair basketball*.

b *Two wheelchair basketball players (premier league) and one wheelchair hockey player (Dutch national team)*.

Based on the obtained data, a machine learning-based classification model was trained to classify for each time instance whether a *small* or *large* correction size is advantageous. This classification was used to extend the MW filter (see section Filter Design). To assess the validity of the resulting extended MW filter, trunk inclination was calculated based on MOCAP data and IMU data processed with the extended MW filter. Also, comparisons with the original (not extended) MW filter were made.

### Equipment

Two IMUs (NGIMU, X-IO Technologies, Colorado Springs, CO, United States) were used to collect 3D inertial sensor data of the trunk and the wheelchair with a sample frequency of ~100 Hz. A 10-camera optoelectric MOCAP system (OptiTrack Prime, NaturalPoint, Inc., Corvallis) with a sampling rate of 120 Hz was used to record the 3D orientation of the segments of interest. The trunk marker cluster frame constituted of four markers connected to a rigid body, and was attached to the sternum (manubrium sterni). The wheelchair marker cluster frame constituted of five markers connected to different positions on the wheelchair frame. The video camera (Casio Exilim, Casio, Tokyo, Japan) recorded the entire track layout with a sample frequency of 60 Hz.

### Wheelchair Sport-Specific Activities

**Figure 1:**
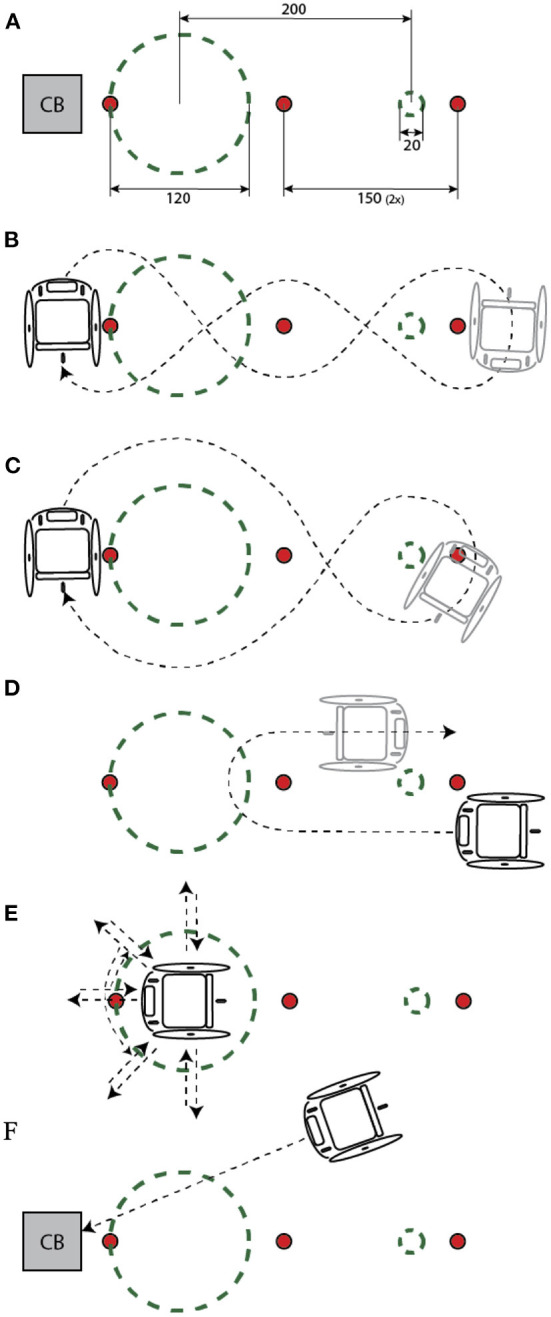
**(A–F)** Track layout with dimensions in cm **(A)** corresponding to the tests as explained in Table 2. Cones and collision block (CB) were removed during test parts in which they were not used. During tests with ‘no’ speed, the wheels of the wheelchair were blocked. This figure was adopted from (van der Slikke et al., 2015).

The wheelchair sport-specific test session is described in Table 2, Figure 1 and covers the main aspects of wheelchair basketball, tennis, rugby, triathlon, and racing. Certain tests were similar to ones used in prior research on wheelchair IMUs (Pansiot et al., 2011; van der Slikke et al., 2015), while tests 1, 10, and 11 in Table 2 were added to put more focus on trunk motion. Prior to the session, the tests were explained, and participants without wheelchair experience were instructed to ride in the wheelchair for ~5 min to familiarize with wheelchair propulsion. The participants were instructed to adopt a neutral pose for at least 20 s at the start and end of the session. All tests were performed in a motion lab.

**Table 2:** All sport-specific tests, together with a description of each test and the speed at which the participants were instructed to perform the test (see also Figure 1). All tests were carried out in immediate succession.

**Test**		**Speed**	**Description**
1	Isolated trunk rotations	No	3x flexion/extension, left/right lateral flexion and left/right axial rotation
2	Straight 5 m	Normal	3x sprint with static trunk
	Straight 5 m	Low	3x
	Straight 5 m	Normal	3x
	Straight 5 m	High	3x
3	Straight skid	High	2x sprint (stop with skidding wheels)
4	Slalom	Normal	Around 3 cones (Figure 1B)
	Slalom	High	Around 3 cones (Figure 1B)
5	8 shape	Normal	(Figure 1C)
	8 shape	High	(Figure 1C)
6	U turn	Normal	180 clockwise turn (Figure 1D)
	U turn	High	180 clockwise turn (Figure 1D)
	U turn	Normal	180 anti clockwise turn (Figure 1D)
	U turn	High	180 anti clockwise turn (Figure 1D)
7	Turn on spot	Normal	360 clockwise turn
	Turn on spot	Normal	360 anti clockwise turn
	Turn on spot	High	360 clockwise turn
	Turn on spot	High	360 anti clockwise turn
8	Star twist	Free	Star wise bi-directional rotation
	Star twist	Free	As previous, combined with back-and-forth movement (Figure 1E)
9	Collision	Free	2 ×2 m sprint and collision against a block of 30 kg (Figure 1F)
10	Tennis movements	No	Do two service- and two backhand motions with tennis racket
11	Ball handling	No	Pick up ball from the ground (2x) and throw ball away with one hand

### Data Pre-processing

#### Pre-processing of MOCAP Data

OptiTrack 3D position data of the frame and trunk markers were acquired in Motive 2.2.0 (Natural Point), converted to a C3D format, and imported in MATLAB (R2019b, The Mathworks Inc., Portola Valley, CA, United States). Missing values were interpolated if the duration of the gap was < 0.2 s and subsequently resampled from 120 to 100 Hz by spline interpolation. Based on the first sample of each time series, the 3D local coordinate frames of the trunk and the wheelchair were determined based on the positions of the three markers with the lowest number of missing values (Kontaxis et al., 2009). The local marker coordinate frames with respect to the global marker coordinate system were tracked over time.

#### Pre-processing of IMU Data

First, the magnetometer (hard iron) offset of the IMU data was corrected (MathWorks, 2020). Subsequently, sample frequency deviations were corrected by resampling the data to 100 Hz by spline interpolation. Given the obtained 3D accelerometer, 3D magnetometer, and 3D gyroscope data, the IMU orientation was determined using the MW filter and the correction size (Madgwick et al., 2011). First, an IMU orientation estimate was obtained using the original MW filter to enable time alignment of the IMU and marker data (see next paragraph). For this, a correction size of 0.033 was used as reported by Madgwick et al. (2011). A grid search for different beta values on the current dataset supported the use of this value. Second, the earth frame-only estimate (without gyroscope data) was obtained, which will be used to generate the classification model (see section Data Analysis). For this estimate, the direction of the acceleration vector was regarded “down” and the direction of the magnetometer vector was regarded “north.”

#### Determining Trunk Inclination

To convert the IMU- and MOCAP-based orientations into a one-dimensional inclination angle between the trunk and the wheelchair, a helical approach was used. First, the rotation matrix between the proximal (wheelchair) segment and the distal (trunk) segment were obtained. Subsequently, this rotation matrix was represented relative to the first static sample, in which the person was positioned in neutral pose. Neutral pose was considered 0 degrees trunk inclination, in which positive values indicate trunk flexion. Accordingly, the helical angles could be calculated (Blankevoort et al., 1990). After obtaining the helical angles of both IMU and MOCAP systems, they were synchronized with respect to time by cross-correlation of the helical angle time series (Rhudy, 2014). After synchronization, the helical angles were determined again to ensure that all orientations were relative to the same static (neutral pose) sample.

### Data Analysis

#### Filter Design

**Figure 2:**
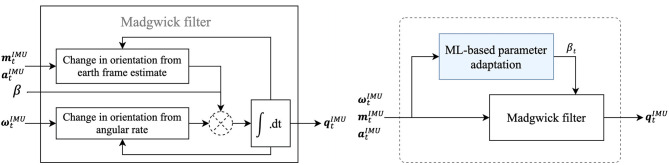
A simplified block diagram of the MW filter (left) and a block diagram of the machine learning (ML)-based extended MW filter (right). Within the extended MW filter, the correction size (ß) may change over time (i.e., ß_t_), which differs from the original application of the MW filter. The filters use IMU data consisting of 3D gyroscope data ($\omega_{t}^{IMU}$), 3D magnetometer data ($m_{t}^{IMU}$), and 3D accelerometer data ($a_{t}^{IMU}$) as input and IMU orientation ($q_{t}^{IMU}$) as output. Note that the white box in the left figure corresponds to the white box in the right figure.

Using the data collected, trunk inclination angles are determined using the original MW filter and the extended MW filter, which are explained in more detail in this section. Figure 2 (left) shows a representation of the original MW filter, which corrects for integration drift on the gyroscope-based estimate (Δ*q*_ω,*t*_) based on the earth frame-based estimate. The earth frame-based correction (Δ*q*_*am,t*_) is determined by a gradient descent algorithm based on the accelerometer and magnetometer data (Madgwick et al., 2011). Subsequently, the correction step is normalized to a pre-determined magnitude, the correction size or ß, such that the resulting orientation at time *t* ($q_{t}^{IMU}$) is calculated according to Equation 1. The correction size is the only parameter to be tuned when using the MW filter.

(1) $$\begin{array}{r} q_{t}^{IMU}=q_{t-1}^{IMU}+\left(\Delta q_{\omega ,t}-\;\beta \;\Delta q_{am,t}\right)\Delta t \end{array}$$

**Figure 3:**
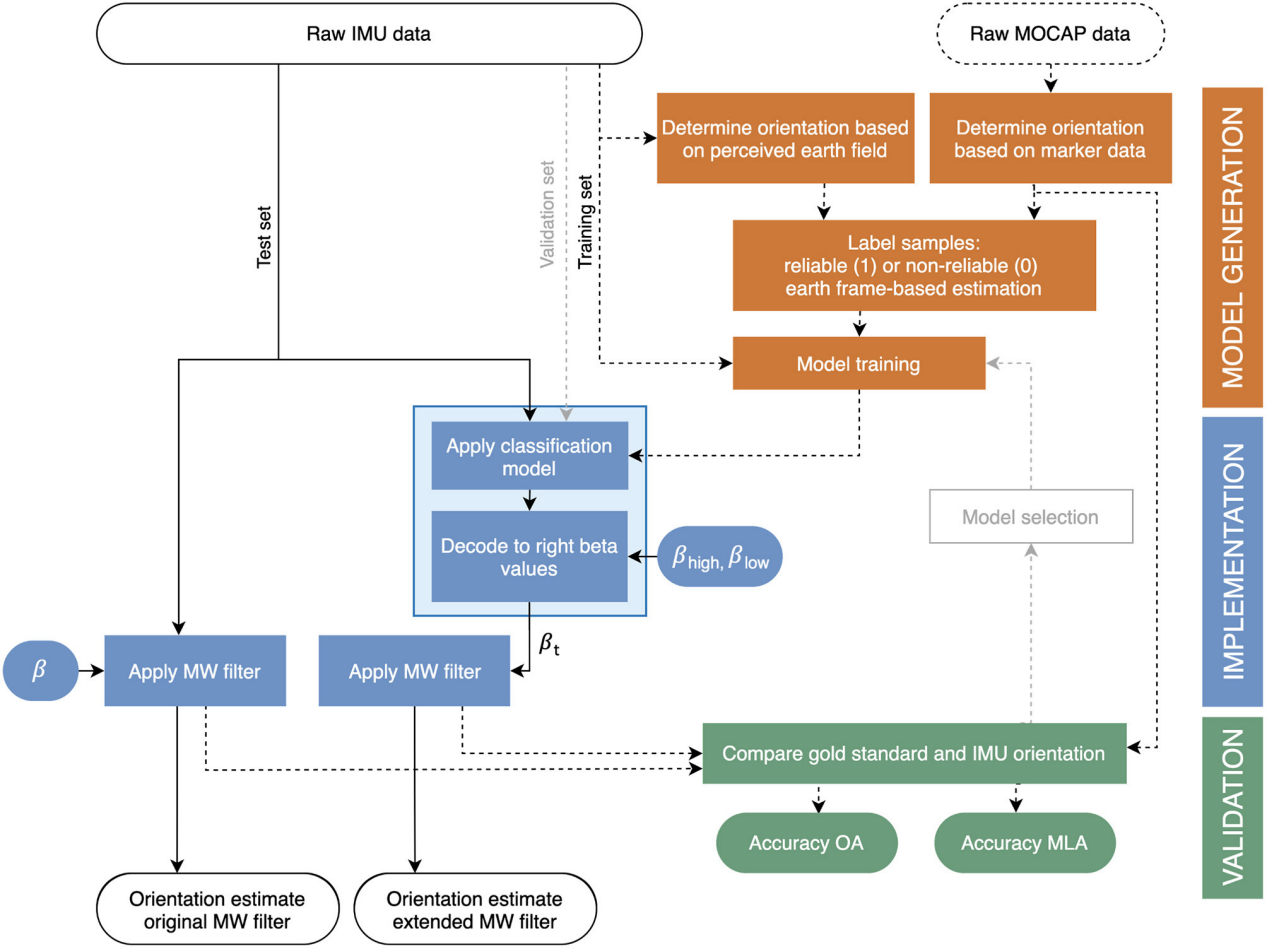
Overview of the model generation, implementation and validation steps, and corresponding in- and outputs in this study. Model implementation for the original MW filter (left blue) is compared with that of the extended MW filter (right blue) as proposed in this study. Model generation and validation is done using MOCAP data, and was performed only once. Those steps are, therefore, indicated by dashed lines (- -). Before the classification model was chosen, several models were trained, implemented, and compared with MOCAP data, after which the best model was selected (see model selection) using data of the validation set. This loop is indicated by gray dashed lines (- -). The solid lines indicate the steps that should be taken each time one wants to estimate the IMU orientation. Note that the light blue box corresponds to the light blue box in Figure 2.

Figure 2 (right) shows the proposed extension of the MW filter. Instead of one correction size (ß), two different correction sizes were determined for the extended filter; a large correction size for situations in which the earth frame-based estimate is expected to be correct, and a small correction size otherwise. To apply each correction size at the right instance in time, the validity of the earth frame-based estimate should be determined based on the raw IMU data (which is the only available data at this point). Therefore, the MW filter was extended with a machine learning-based classification model that predicts whether or not the earth frame-based estimate will be correct based on IMU data. Accordingly, this prediction will be used to adapt the correction size (ß) to a high value when a correct earth frame-based estimate was predicted (i.e., ß_high_) and to a low value (i.e., ß_low_) otherwise. This “decoding” results in a correction size for each instance in time, i.e., ß_t_, which is an input of the MW filter (see Figure 2). Figure 3 shows a step-by-step explanation of model generation, implementation, and validation.

#### Model Generation

##### Label Samples

To generate the classification model, training samples were created with known input (raw IMU data) and output (labeled *correct* (Camomilla et al., 2018) or *in-correct* [0] earth frame-based orientation estimate) data. These output labels were obtained by comparing the “earth frame-only” orientation estimate with the MOCAP-based orientation on a sample-to-sample basis. A sample was labeled “correct” (i.e., 1) if the difference between the “earth frame-only” and the MOCAP-based trunk inclination was < 1 degree + noise, and was labeled “incorrect” (i.e., 0) otherwise. To determine noise, the standard deviation of the difference between the MOCAP-based inclination and the earth frame-only estimate during 20 s neutral pose were assessed for all the participants and accordingly averaged. The labels were saved as “EFcorrect.”

Labeling this way may cause some samples to be falsely labeled “correct” because of coincidental intersections between the MOCAP system and a deviating earth frame-only estimate. Therefore, a second, more conservative outcome variable was defined in which a sample is labeled “correct” only if the maximal difference of five consecutive samples (that sample, two preceding samples and two following samples) was < 1 degree + noise. These labels were saved as “EFcorrect_S5.” The labels of “EFcorrect” and “EFcorrect_S5” were determined for all samples and added to the dataset.

##### Model Training

First of all, the participants were divided into a training set, a validation set, and a test set (with six, three, and two participants, respectively, see Figure 3). The training set included one elite, one active, and four non-experienced wheelchair users, the validation set included two active and one non-experienced wheelchair user, and the test set included two elite wheelchair athletes. Subsequently, all data were imported in Python (version 3.7, Python Software Foundation, Wilmington, DE, United States) to perform machine learning. Before learning, all input data were standardized by z-normalization (Goldin and Kanellakis, 1995), and the training data were balanced (by randomly removing samples of the majority class) such that the number of samples labeled 1 was equal to that labeled 0 (Pedregosa et al., 2011). Accordingly, the 18 features (two sensors with each 3D accelerometer, magnetometer, and gyroscope [2^*^3^*^3]) were ranked on importance by recursive feature selection on a random forest classification algorithm (Pedregosa et al., 2011) with a leave-one-subject-out cross-validation (LOSO-CV) on the training set (Pedregosa et al., 2011). By LOSO-CV, the model is trained on all-but-one participant of the training set, and accordingly evaluated on the participant that was left out. Subsequently, the feature ranking was used to select the best number of features. The five best sets (based on precision, recall and F1-score of the LOSO-CV) were selected for further model training.

To determine which learning algorithm is the most suitable, four different classification algorithms were trained. Since the data are tabular and relations are expected to be non-linear, a Gaussian Naive Bayes algorithm, a logistic regression, a decision tree algorithm, and a random forest algorithm were compared (Pedregosa et al., 2011). Also, the two different outcome variables (EFcorrect and EFcorrect_S5) were compared. Since five sets were left from the feature selection procedure, 40 models (four learning algorithms, two outcome variables, and five sets of features) were trained.

#### Implementation

##### Decode to **β**_**t**_ and Apply MW Filter

After applying a model, the outcome matrices that consist of predicted 1's (correct earth frame-based estimation) and 0's were decoded to ß_high_ and ß_low_, respectively. Subsequently, the IMU orientations were calculated by the MW filter, and the helical angles were determined (see Figure 3). The best values for ß_high_ and ß_low_ were chosen by applying the extended MW filter on the EFcorrect labels of the training and validation set. All combinations of ß_low_ from 0 to 0.01 (steps of 0.001) and ß_high_ from 0.5 to 1 (steps of 0.025) were applied. The RMSE between the IMU-based trunk inclination and the MOCAP-based trunk inclination was used to determine the final values for ß_high_ and ß_low_.

##### Model Selection

After training all the 40 models, the best model was selected by comparing the models on the validation data. Therefore, all the models were fit to the validation data, and trunk inclination angles were determined. The best model was selected based on the lowest mean absolute error (MAE) and RMSE between the IMU-based angles and the MOCAP-based angles. To evaluate the performance of the final classification model, precision, recall, and accuracy were reported. Also, the hyperparameters of the particular algorithm were tuned using by random search LOSO-CV on the training set. Subsequently, the final model was trained on the training set and was implemented on the test set to assess its performance.

#### Validation

To determine the accuracy of trunk inclination based on the extended MW filter and to determine the difference between the extended and the original MW filter, the mean error, RMSE, and MAE with respect to the MOCAP-based inclination angles were determined for both filters. Also, the correlation between trunk inclination determined using MOCAP data and the trunk inclination determined using the IMU data with both filters was determined.

To compare the filters for activities with different levels of dynamics, a distinction was made between “slow to moderate sprints” (Table 2.2 with speeds “normal” and “low”), “fast sprints” (Tables 2.2, 2.3 with speed “high”) and “agility exercises” (Tables 2.4–2.9). The parts were selected manually using the video frames. For each of the three parts, MOCAP-based trunk inclination was plotted against the original and extended MW filter-based trunk inclination to assess eventual angle dependencies. Also, a Bland–Altman analysis (Bland and Altman, 1986) was performed on the three parts to compare mean differences and 95% confidence intervals between MOCAP-based trunk inclination angles and those determined by the extended MW filter. To compare situations in which the wheelchair was fixed to the ground, i.e., “fixed wheelchair” part, and in which it was not, sprints and agility exercises were taken together to represent the “free wheelchair” part. Mean error, MAE, and RMSE between both filters and the MOCAP-based trunk inclination were determined for both the fixed wheelchair part and the free wheelchair part. In addition, the evolution of trunk inclination over time was presented for isolated trunk rotations (Table 2.1). in the fixed wheelchair part and for both star twists (Table 2.8) in the free wheelchair part.

To gain more insight into the behavior of the machine learning model, an analysis was performed of the situations in which small and large correction sizes were applied and their durations.

## Results

Eleven participants were included (six in training set, three in validation set, two in test set) with a mean session duration of 14.6 min. Of this, 14.4% of the samples was labeled 1 (<2.27° difference with MOCAP-based trunk inclination) according to the criteria as defined for “EFcorrect,” and 12.5% of the samples were labeled 1 for “EFcorrect_S5.” After balancing, the training set consisted of 155,032 and 133,426 samples for EFcorrect and EFcorrect_S5, respectively, with equally represented labels.

### Implementation

Applying the extended MW filter on labeled data for different values for ß_high_ and ß_low_ yielded the smallest RMSEs with the reference system when ß_high_ ranged from 0.925 to 1, and when ß_low_ ranged from 0 to 0.003. Therefore, the mean of these values was taken such that ß_high_= 0.9635 and ß_low_ = 0.0015.

Based on feature selection, the final set of features consisted of *a*_*x, trunk*_, *m*_*y, trunk*_*m*_*x, trunk*_, *m*_*z, wheelchair*_, *and m*_*x, wheelchair*_, in which *x* represents the sagittal axis (forward-backward), *y* represents the transversal axis (left-right), and *z* represents the longitudinal axis (up-down). Using this feature set, the models were trained and implemented to determine trunk inclination. The IMU-based trunk inclination based on the different models were compared with the MOCAP-based trunk inclination (see Table 3). The smallest difference with the MOCAP-based trunk inclination was found for the fandom forest classification with label “EFcorrect.” Compared with the labeled data, this model showed a precision, recall, and accuracy of 0.9, 0.95, and 0.86, respectively.

**Table 3:** Performance of the eight models left after selecting the final set of features in terms of mean absolute error (MAE) and root-mean-squared error (RMSE) between MOCAP-based trunk inclination and the trunk inclination of the extended MW filter based on validation data.

**Classification** **algorithm**	**MAE (°)**		**RMSE (°)**	
	**EFcorrect**	**EFcorrect_S5**	**EFcorrect**	**EFcorrect_S5**
Decision tree	9.6	9.2	13.3	13.4
Random forest	8.9	10.3	13.6	15.8
Naive bayes	14.2	14.4	23.5	24.0
Logistic regression	11.7	12.0	18.7	18.9

### Validation

To gain more insight into the performance of the original and the extended MW filters, comparisons were presented for parts in which the wheelchair could move (free wheelchair) and could not move (fixed wheelchair), separately (see Table 4). Results indicate that the extended MW filter outperforms the original MW filter and performs particularly better during “free wheelchair” instances (MAE decreased from 9.5 to 5.9° and RMSE from 11.7 to 7.6°, on average) with improvements by up to 47% MAE and 45% RMSE. During “fixed wheelchair” instances, the models show equal performances with average RMSEs of 6 and 5.3° for the original and the extended MW filters, respectively. The extended MW filter showed correlations of 0.86 (fixed wheelchair) and 0.92 (free wheelchair) with the MOCAP data, which were stronger than those of the original MW filter in all situations.

**Table 4:** Comparison of the mean error, mean absolute error (MAE), root-mean-squared error (RMSE), and correlation (r) between the MOCAP data and the original MW filter and between the MOCAP data and the extended MW filter.

**Condition**	**MW filter**	**Mean error (°)**		**MAE (°)**		**RMSE (°)**		**r**	
		**S1**	**S2**	**S1**	**S2**	**S1**	**S2**	**S1**	**S2**
Fixed wheelchair	Original	−1.3	0.3	4.5	4.3	5.5	6.5	0.88	0.95
	Extended	0.0	2.2	4.1	4.0	5.2	5.4	0.88	0.97
Free wheelchair	Original	−8.2	−5.5	10.2	8.8	12.4	11.0	0.72	0.80
	Extended	1.6	2.7	5.4	6.5	6.8	8.4	0.87	0.86

*Results were presented for parts in which the wheelchair could not move (fixed wheelchair), and for parts in which the wheelchair could move (free wheelchair) for subject 1 (S1) and subject 2 (S2) of the test set*.

**Figure 4:**
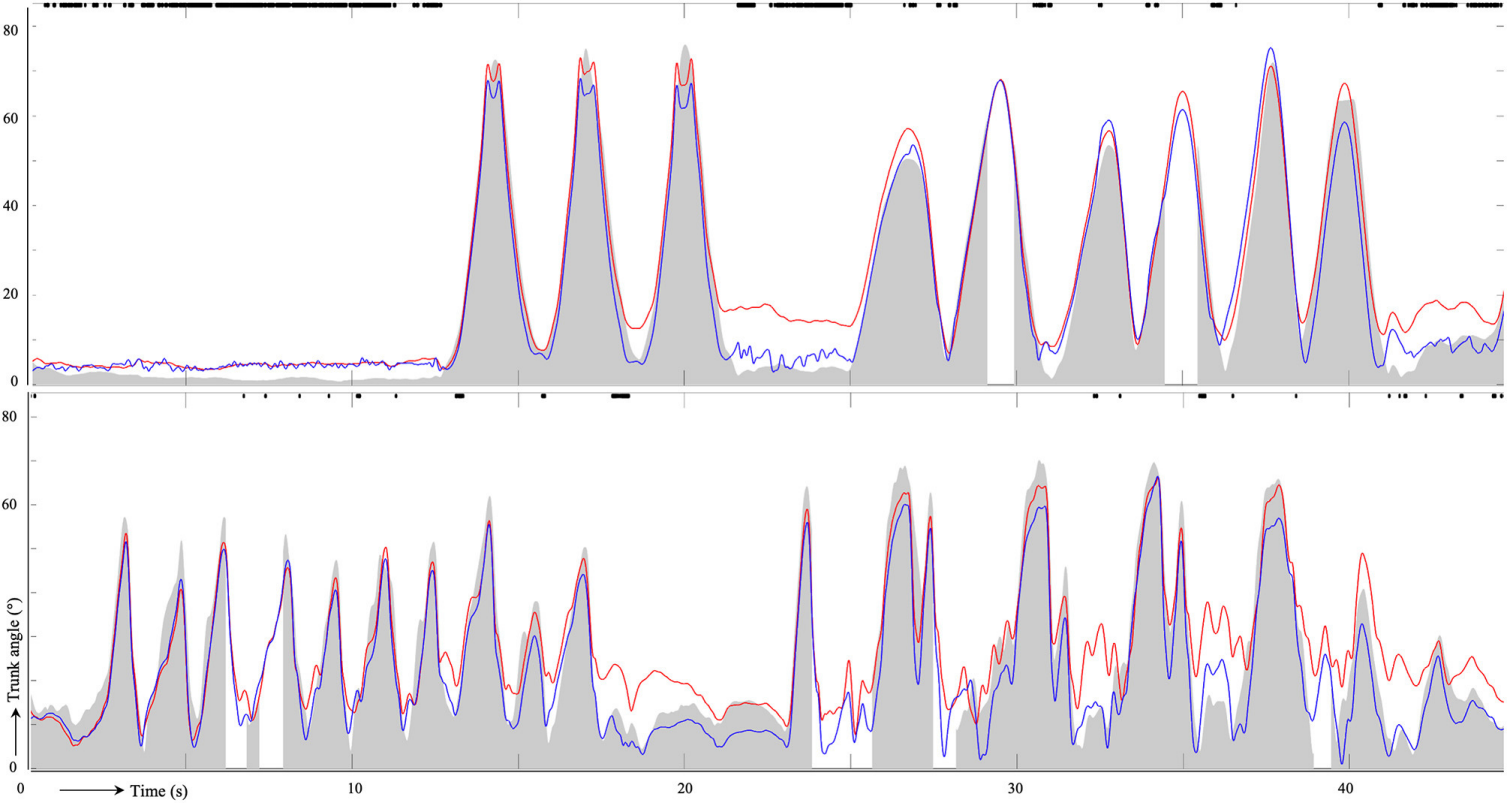
Typical plots of the trunk inclination angles over time of the original MW filter (red) and that of the extended MW filter (blue) for the isolated trunk rotations in a static wheelchair, i.e., Test 1, (upper figure) and star twists in a free wheelchair, i.e., Test 8 (lower figure). The MOCAP-based trunk inclination is indicated by the gray surface (which is interrupted at some time frames because of insufficient marker visibility). The black dots indicate time instances at which ß_high_ was applied. The data was from one of the test subjects, a three-point elite wheelchair basketball athlete.

**Figure 5:**
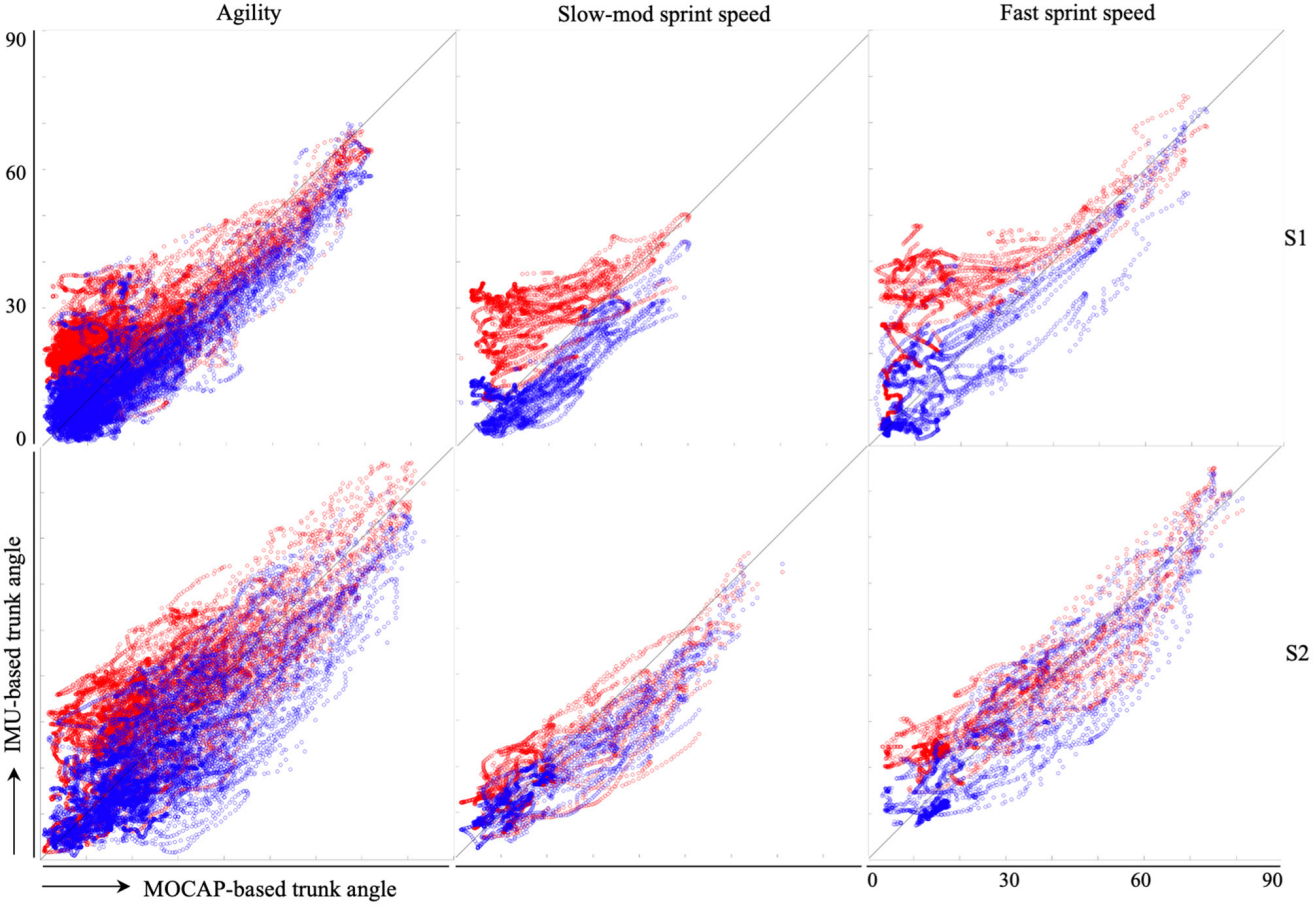
Scatter plots of the inclination of the original filter (red) and that of the extended filter (blue) against the MOCAP-based trunk inclination for the “agility” part (left), “slow-mod sprint speed” part (middle) and “fast sprint speed” part (right) of subject 1 (upper three figures) and 2 (lower three figures) in the test set.

Figure 4 shows the trunk inclination of the original (blue) and the extended MW filters (red) against the reference system for isolated trunk rotations and startwists (Tables 2.1, 2.8). Instances in which ß_high_ was applied are indicated by the black dots in Figure 4. Figure 5 shows the trunk inclination of both filters against the MOCAP-based inclination for three “free wheelchair” parts of the session. Bland–Altman analyses reveal a mean difference of 3.5° for slow to moderate sprints, 1.4° for fast sprints, and 2° for agility exercises between extended MW filter-based and MOCAP-based trunk inclination. The corresponding 95% limits of agreement were −7.6 and 14.6° (slow to moderate sprints), −12.3 and 15.2° (fast sprints), and −13 and 16.9° (agility exercises).

To gain more insight into the behavior of this model, the time instances in which large or small correction sizes were applied were analyzed. The black dots in Figure 4 indicate that ß_high_ was most common in static situations, while ß_low_ was most common in dynamic situations. The duration of successive ß_low_-instances had a median of 0.05 and 0.03 s for subjects 1 and 2 in the test set, respectively, and ranged from 0.01 to 15.3 s for subject 1 and from 0.01 to 2.05 s for subject 2. The median duration of successive ß_high_-instances was 0.03 and 0.05 s for subjects 1 and 2, respectively, and duration ranged from 0.01 to 0.99 s for subject 1 and from 0.01 to 34.3 s for subject 2.

## Discussion

The aim of this study was to explore whether machine learning-based classification could be used to extend the MW filter to make it applicable for highly dynamic situations (where assumptions on earth-frame based estimates are invalid). We specifically studied the proposed algorithms for estimating the instantaneous trunk inclination in wheelchair sports. Results indicate that the extended MW filter performs better than the original MW filter in assessing instantaneous trunk inclination (7.6 vs. 11.7° RMSE), especially during the dynamic IMU-challenging situations with moving athlete and wheelchair. Compared with the extended MW filter, the difference between the original filter and the reference system increased for lower trunk inclination angles. This might be because of the slight underestimation of large trunk inclination angles by the IMU-based approaches, such that deviations due to drift are mainly visible at lower inclination angles, while they diminish at higher angles.

To the knowledge of the authors, no previous studies investigated the accuracy of IMU-based body segment orientation during wheelchair activities or other dynamic IMU-challenging situations. Therefore, only trunk inclination accuracy of the fixed wheelchair parts, i.e., the less dynamic parts, allows for comparison with previous studies. The 5.3° RMSE for trunk inclination in this study is comparable with the trunk inclination accuracy of 5° RMSE during postural disturbances when walking on a treadmill (Miller and Kaufman, 2019) and the 3–4.9° RMSEs for the estimation of trunk orientation during dynamic sports motions with both legs on the ground (Brouwer et al., 2020). Although the latter results were more accurate, the measurement duration was much shorter (<30 s Brouwer et al., 2020) than the session durations in this study (~15 min). Also, some studies reported somewhat better accuracies of the IMU-based estimation of body segment orientations (Mazzà et al., 2012; Bergamini et al., 2013; Shull et al., 2017), but those studies measured for very short periods (Bergamini et al., 2013) or studied tasks with a small range of motion (Mazzà et al., 2012; Shull et al., 2017). Of the mentioned studies, all studies reported a Kalman filter (Mazzà et al., 2012; Bergamini et al., 2013; Shull et al., 2017; Miller and Kaufman, 2019) or a MW filter (Brouwer et al., 2020) as AHRS, and all studies validated the IMU-based estimations using an optical motion capture system. Overall, it can be concluded that the better performance of the method of the authors for highly dynamic situations has not been gone at the cost of a lower accuracy than that of previously reported results during less dynamic situations.

Since, to the knowledge of the authors, trunk inclination accuracy during highly dynamic sports situations is not reported in the literature yet, comparing the results of the original and the extended MW filters of this study may provide more insight. A major difference between the filters is their performance during dynamic situations; the extended MW filter performed better than the original MW filter in the dynamic “free wheelchair” situations. This can be explained by the extent to which accelerations were present in these situations. When the wheelchair is propelled, continuous accelerations and decelerations are present, and the magnitude of accelerations is determined by the acceleration of the wheelchair plus the acceleration of the trunk relative to the wheelchair. Therefore, the earthframe-based estimate is wrong relatively often during “free wheelchair” instances causing the original filter to deviate, while the extended MW filter remains accurate by reducing the impact of this estimate. A similar trend was obtained from the time evolution of IMU-based trunk inclination in which the filters perform equally well in long-term static pose, while the original MW filter shows an increasing deviation during dynamic instances. Moreover, during static instances following dynamic instances, the extended MW filter immediately increases the correction size to “reorient” toward the earth frame-based estimate such that accumulated drift is corrected at once. For the original filter, this “reorientation” takes much longer, which may be a second explanation for the larger errors observed at smaller inclination angles for the original MW filter.

From the previous paragraphs, it may be concluded that the extended MW filter provides considerable improvements compared with the original MW filter. Crucial for this performance is the machine learning-based classification model. To gain more insights into the behavior of this model, the time instances at which large or small correction sizes were applied were analyzed. As expected, ß_low_ was most common in dynamic situations such that the orientation estimate was hardly affected by (wrong) earth frame-based estimates, whereas ß_high_ was most common in static situations such that the effect of drift was limited. Since the orientation estimates during ß_low_ instances rely mainly on integrating the gyroscope signal, integration drift will accumulate for each successive ß_low_ instance. Therefore, the duration of successive ß_low_ instances should not be too long. According to the durations observed in this study (maximum durations of 15.3 and 2.05 s with only two occurrences >10 s), it is assumed that drift was corrected before it may have caused any noteworthy deviations. In general, this relatively simple machine learning model seems to predict the most “advantageous” correction size in each instance well and seems suitable for AHRS extension. Although the MW filter was used in this study, applying this extension to other orientation estimation filters may be promising as well.

Although the extended MW filter performed better compared with the original MW filter, the question whether the method can be seen as sufficiently accurate depends on its application and the aim that is to be accomplished. McGinley et al. (2009) performed a systematic review on the reliability of 3D kinematic gait measurements with regard to clinical interpretation. According to McGinley et al. (McGinley et al., 2009), errors below 5° will be widely considered acceptable to reasonable, while errors exceeding 5° may mislead interpretation. Since gait motions considerably differ from body motions during wheelchair propulsion and the range of trunk motion during wheelchair sports will exceed that of body segments during clinical gait, the minimal acceptable error may be somewhat higher in wheelchair propulsion. Considering the 7.6° RMSE obtained here and a range of motion of 70–80 degrees (see Figures 4, 5) during wheelchair propulsion, the system should be able to differentiate trunk inclinations higher than 11% of the range of motion. For application in wheelchair sports, trunk inclination angle can be used to approximate the center of mass displacement or to analyse motion patterns. A RMSE of 7.6° or 11% is expected to have an effect on above-mentioned analyses of trunk inclination only to a limited extent and will, therefore, be regarded acceptable.

In this study, external validity had priority above acquiring the smallest possible error, and it was aimed to avoid any unnecessary complexity, such that sports scientists will be able to implement the extended MW filter with limited effort. In this regard, some choices were made that may have influenced the final results. Some examples that might have produced more accurate results are (1) measuring all participants in the same wheelchair instead of using their own wheelchair, (2) compensate for magnetic distortions in the motion lab by performing a specific “mapping” of the laboratory (de Vries et al., 2009), (3) start a new measurement for each specific exercise, (4) optimize ß_high_ and ß_low_ after training the final model, and (5) add more refinement in ß values, instead of only ß_high_ and ß_low_. To put the focus on external validity and repeatability, these examples were not applied. Therefore, we expect the results to be well-translatable and implementable to sports and rehabilitation practice.

### Limitations

Although this study provided useful outcomes with regard to orientation estimation in dynamic sports situations, some limitations should be noted. First of all, the trunk markers that were used to determine the MOCAP-based trunk inclination were placed on the upper sternum (to ensure visibility), while the trunk IMU was attached to a lower location on the sternum using a chest strap (to enhance reproducibility and usability and to limit skin artifacts). Since the sternum is rigid, the use of different locations was assumed to have no effect on the measurements. Second, in this study, helical angles were used to determine trunk inclination during wheelchair sports activities. Therefore, caution should be exercised in generalizing the results of this study to situations in which trunk motion is analyzed in terms of anatomical angle definitions. Third, a relatively low number of subjects was included in this study. However, since the results of the measurements with the subjects in the test set showed the same trends and was based on over 100,000 samples, similar results are expected to be obtained when a larger sample of subject was included.

### Future Perspectives

For measuring trunk inclination in wheelchair sports, the machine learning-based extended MW filter is ready for use. Since differently skilled participants were used in this study, it may be assumed that the extended MW filter works well for trunk inclination estimation in all types of wheelchair users, for all types of wheelchairs, and in both rehabilitation as well as (elite) sports practice. Measuring trunk inclination during on-site wheelchair sports offers many opportunities. When trunk inclination is combined with wheelchair kinematics during wheelchair sports (van der Slikke et al., 2015), the (simplified) kinematic state of the wheelchair-athlete combination can be obtained. In this way, center of mass displacement can be approximated, and field-based power losses and power production can be more accurately obtained than based on the wheelchair motions only. This enables more insight into training load, fitness, and the effect of different push techniques. Also, information about trunk inclination can be fed back directly to the coach and/or athlete for specific training purposes.

For application in other IMU challenging sports situations, it is expected that the extended MW filter will also work. From the raw IMU data, only two simple additional steps, (1) run the classification model on the raw IMU data (*a*_*x, distal segment*_, *m*_*y, distal segment*_, *m*_*x, distal segment*_, *m*_*z, proximal segment*_, *m*_*x, proximal segment*_), and (2) decode the outcomes to ß_high_ and ß_low_ (0.9635 and 0.0015 in this study), have to be performed to convert the original MW filter into the proposed extended MW filter. These steps are schematically represented on the left side of Figure 3. After obtaining ß_t_, the MW filter can be executed such that IMU-orientation is obtained. The approach to determine the best values for ß_high_ and ß_low_ is equal to that in the original MW filter (Madgwick et al., 2011) and may differ between sensors and situations. Although the classification model should be verified for other sports, it is expected to be transferable to other situations since (1) the model was based on raw IMU data only and (2) works on a sample-to-sample basis such that differences in movement pattern should not cause any problems. If only one IMU was used, or accelerations in multiple directions (relative to the IMU) were common, a custom-made random forest classification model is recommended for optimal results.

## Conclusion

The extended MW filter with machine learning-based classification improved orientation estimation in sports applications that are challenging for IMU usage. The extended MW filter resulted in accurate trunk inclination angles during wheelchair sport-specific exercises. During exercises in which the wheelchair was moved unrestrictedly, the extended MW filter performed better than the original MW filter. During situations in which the wheelchair was static (by blocking the wheels), both the original and the extended MW filters performed equally well. In conclusion, the extended MW filter is a promising application for the estimation of body segment orientation using IMUs in highly dynamic sports situations and is ready to be used in (elite) wheelchair sports and rehabilitation practice.

## Data Availability Statement

The raw data supporting the conclusions of this article will be made available by the authors, without undue reservation.

## Ethics Statement

The studies involving human participants were reviewed and approved by Human Research Ethics Committee (HREC) of Delft University of Technology. The patients/participants provided their written informed consent to participate in this study.

## Author Contributions

MD and MK contributed to conception and design of the proposed orientation estimation algorithm (extended filter). MD, MH, MB, and DV contributed to the conception and design of the experiments. MD performed the measurements, organized the database, performed the statistical analysis, and wrote the first draft of the manuscript. MD, MK, MH, MB, and DV reviewed the manuscript. All authors contributed to manuscript revision, read, and approved the submitted version.

## Conflict of Interest

The authors declare that the research was conducted in the absence of any commercial or financial relationships that could be construed as a potential conflict of interest.

## Publisher's Note

All claims expressed in this article are solely those of the authors and do not necessarily represent those of their affiliated organizations, or those of the publisher, the editors and the reviewers. Any product that may be evaluated in this article, or claim that may be made by its manufacturer, is not guaranteed or endorsed by the publisher.

## Supplementary Material

The classification model to execute the extended MW filter as mentioned in the present study is available via https://doi.org/10.4121/14883927.v1.
