# Supplementary figures and images for: Machine Learning to Improve Orientation Estimation in Sports Situations Challenging for Inertial Sensor Use
Source: Front Sports Act Living. 2021 Aug 3;3:670263. doi: 10.3389/fspor.2021.670263 (PMC8369156; doi:10.3389/fspor.2021.670263)

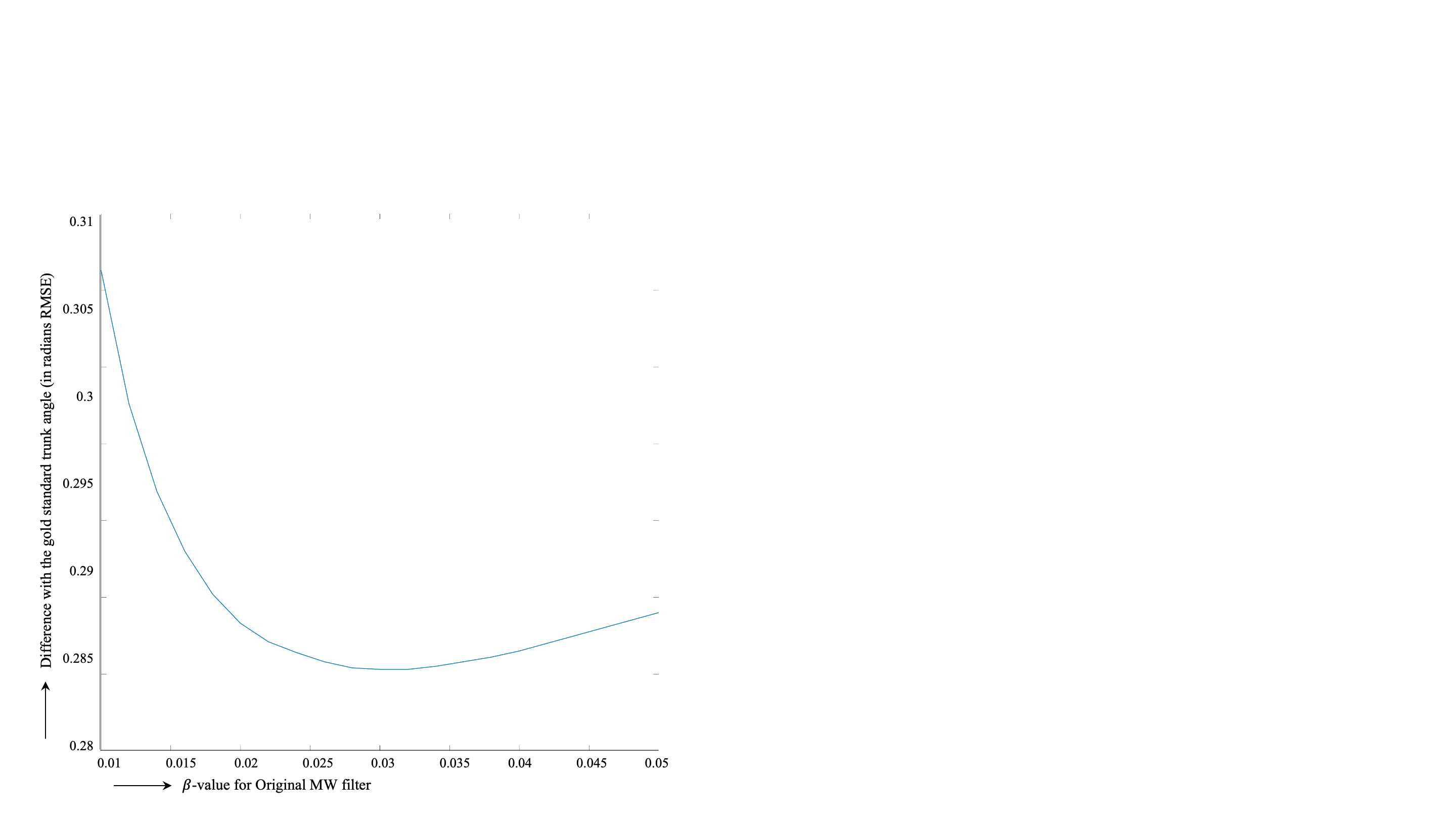

Supplement: Supplementary file 12 [file Image_1.TIFF]

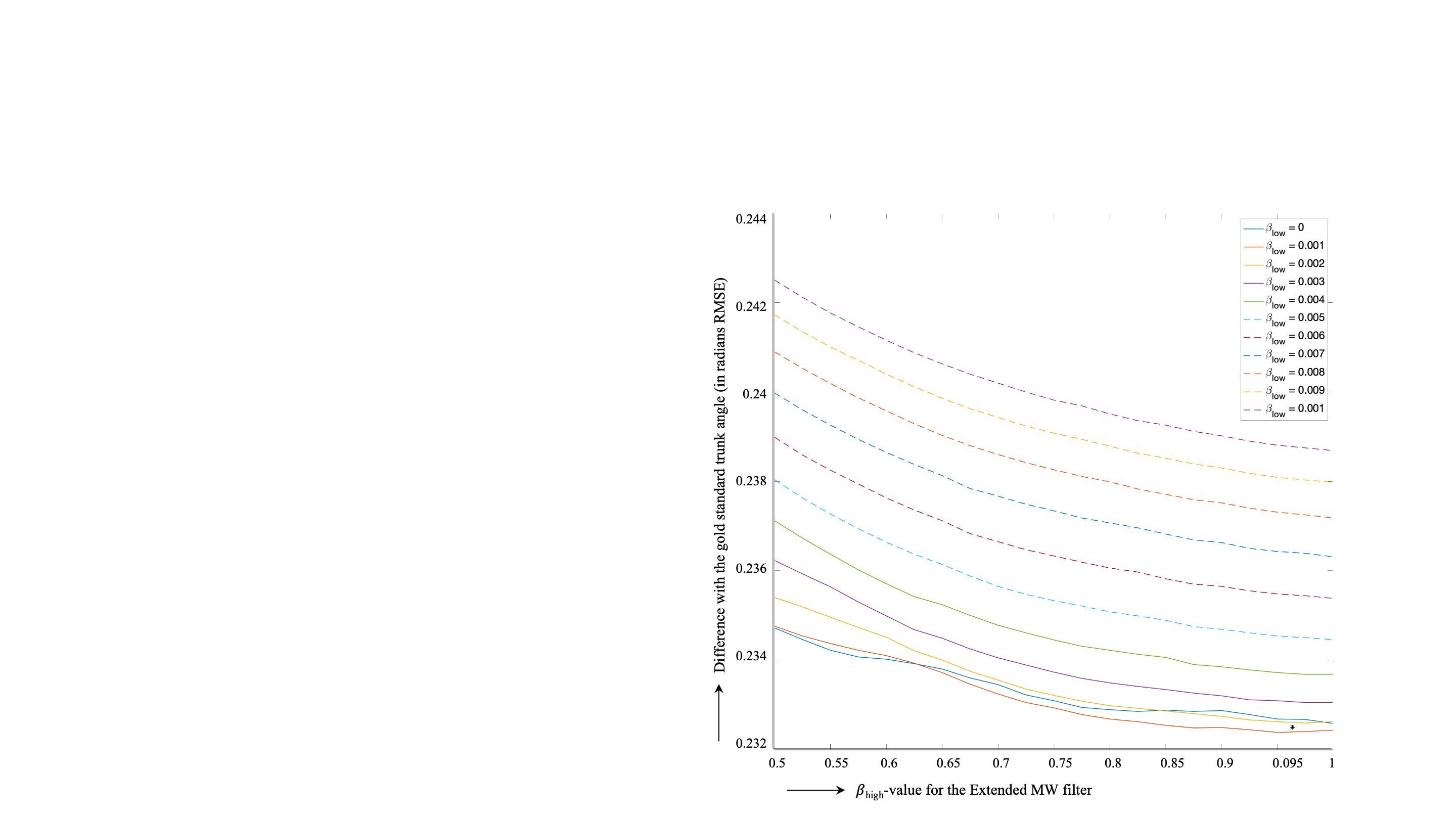

Supplement: Supplementary file 13 [file Image_2.TIFF]
